# Galectin-1 and galectin-3 in male reproduction - impact in health and disease

**DOI:** 10.1007/s00281-024-01032-7

**Published:** 2025-01-02

**Authors:** Monika Fijak, Hiba Hasan, Andreas Meinhardt

**Affiliations:** Institute of Anatomy and Cell Biology, Hessian Centre of Reproductive Medicine, Justus-Liebig University Giessen, Aulweg 123, 35392 Giessen, Germany

**Keywords:** Galectin-1, Galectin-3, Testis, Epididymis, Immune privilege

## Abstract

The formation and differentiation of mature, motile male germ cells, which can fertilize the egg and ensure successful implantation and development of a healthy embryo, are essential functions of the testis and epididymis. Spermatogenesis is a complex, multistep process that results in the formation of motile haploid gametes, requiring an immunoregulatory environment to maintain tolerance to developing neo-antigens. Different cell types (Sertoli cells, macrophages), immunoregulatory factors and tolerance mechanisms are involved. In this context, possible effects of galectins on the immunoregulatory functions and fertilization ability of male germ cells are postulated. Galectins are pleiotropic lectins involved in the homeostasis, modulation of immune responses and pathological processes. Despite the well-recognized role of galectins in female reproduction, the functions of galectins in the male reproductive organs, particularly the testis and epididymis, remain largely unexplored. Among the galectins, galectin-1 and galectin-3 are the best-studied in these organs. This review summarizes the current knowledge of the cellular expression and the roles of galectin-1 and galectin-3 in testis and epididymis and discusses their functions in spermatogenesis, steroidogenesis, epididymal maturation of spermatozoa and inflammatory response.

## Male reproductive system - physiology of testis and epididymis

The testis has two primary functions: producing spermatozoa (spermatogenesis) and synthesizing testosterone (steroidogenesis). These processes occur in separate compartments: spermatozoa develop in the seminiferous tubules with Sertoli cells as ‘nurse cells’, while Leydig cells in the interstitial space produce testosterone. Besides the Leydig cells, the interstitial compartment contains fibrocytes (sparse in mouse, frequent in men), blood and lymphatic vessels and leukocytes. These consist mostly of different subpopulations of macrophages, while few T cells and dendritic cells are also present in addition to mast cells in human. Leydig cells produce testosterone as the main androgen and a plethora of other factors. They depend on LH for normal function and in men can also be found scattered in the tunica albuginea. Each testis is encased in a fibrous capsule, the tunica albuginea, which in men extends inward to divide the testicular parenchyma into about 350 lobules, each containing several seminiferous tubules. The rodent testis is not divided by septae.

The germinal compartment within the seminiferous tubules consists of the somatic Sertoli cells as the main structural framework of the seminiferous epithelium. Sertoli cells provide physical support as well as essential nutrients and growth factors for germ cells to develop. They form tight junctions creating the blood-testis barrier (BTB) that segregates the seminiferous epithelium into a basal and an adluminal compartment. The BTB restricts molecule passage between the interstitial and basal compartment on one side and the adluminal compartment on the other side generating a unique environment for germ cell development. Moreover, Sertoli cells remove residual cytoplasm during transformation of round spermatids to the elongated spermatozoa (spermiogenesis) and secrete tubular fluid that helps transport immotile spermatozoa to the epididymis after release from the Sertoli cells to the lumen (spermiation). Sertoli cells respond to hormonal signals (FSH, testosterone) that regulate spermatogenesis. By these means, Sertoli cells ensure the proper development and transport of spermatozoa within the testis. The latter is assisted by the myoid peritubular cells which surround the ducts in one (rodents) or several layers (men) [1].

Spermatogenesis is a multistep process by which spermatozoa are produced from spermatogonial stem cells. Spermatogonial populations including stem cell spermatogonia are located on the basal layer of the seminiferous tubules, where they undergo mitotic divisions to produce more spermatogonia and finally primary spermatocytes. Primary spermatocytes enter the first meiotic division to produce secondary spermatocytes, which quickly undergo the second meiotic division to form round spermatids. These haploid cells encounter drastic morphological changes to become elongated spermatids. Alterations include the condensation of DNA, formation of the acrosome from Golgi precursors, the development of the sperm flagellum/tail with mitochondrial rearrangement around it to provide energy for movement in the female reproductive tract and shedding of excess cytoplasm in the form of the residual body, which is subsequently phagocytosed by Sertoli cells. Spermatogenesis occurs as a continuous cyclical process, where species dependent different cellular composition and maturation steps of germ cells define so-called stages (mouse stage I-XII, rat stage I-XIV, human stage I-XI). For example, in stage VIII in mouse, spermiation occurs, where elongated spermatids are finally released as spermatozoa from the apical tip of the Sertoli cells into the lumen of the seminiferous epithelium [1]. The latter connect to the rete testis, which extend as efferent ducts to the epididymis.

The efferent ducts drain into the single highly convoluted epididymal duct, where spermatozoa are stored within the lumen, thereby undergoing a complex series of biochemical modifications essential to reach fertilization potential as mature spermatozoa [2]. Development and function of the epididymis depends on the luminal supply of testosterone, which is bound to a carrier protein, the androgen-binding protein (ABP), a secretory product of the Sertoli cell. The epididymis consists of three parts: the head (caput) which contains the distal efferent ducts and the beginning of the epididymal duct, the body (corpus) and the tail (cauda), which continues into the deferent duct. In human, the caput epididymidis consists mainly of the efferent ducts, while in mice the caput also harbors the initial segment not found in men. The epididymis is covered by a thin tunica albuginea and attached to the testis by two small ligaments. Histologically, the epididymal duct is lined with a pseudostratified stereociliated columnar epithelium characterized by various cell types such as principal cells (secretory and resorptive capacity), clear cells (acidification of the luminal fluid), basal cells (stemness) and tight junctions that form a diffusion barrier. The duct’s muscular coat becomes thicker toward the deferent duct [3]. The interstitium consists of blood and lymphatic vessels and fibrocytes that can form thin septae in mouse, but are less clear pronounced in men. Immune cells, mostly macrophage populations, are seen intraepithelially and interstitially [4].

## Testicular immune environment

Auto-antigenic germ cells are formed long after the establishment of immune tolerance and are protected from a deleterious immune response by several complementary defense mechanisms that exist at structural, cellular, molecular as well as environmental levels. These defense mechanisms are in place to preserve and protect the auto-immunogenic germ cells from detrimental inflammatory responses and to regulate the immunoprivileged status of the testis [5, 6].

The testis is an immune privileged organ where experimentally introduced antigens are tolerated without eliciting an inflammatory immune response. As examples, when transplanted in intratesticular locations, allografts and xenografts such as skin, parathyroid glands and adrenal glands survived for considerably long times [7–10]. This immune privileged status is established by several mechanisms. Here, Sertoli cells provide the first level of protection for germ cells by establishing the BTB, which sequesters the germ cells in the adluminal compartment from immune attack. Originally assumed to be the only player in shielding and protecting the germ cells from the immune system, it was later found that sequestration of germ cell neo-antigens is only partial as they can be found already on preleptotene spermatocytes which have not yet passed the BTB, unlike later primary spermatocytes [11]. Antigens of germ cells in the adluminal compartment are phagocytosed by Sertoli cells, which then move and egress basally by transcytosis. This process is crucial for inducing systemic tolerance via regulatory T cells [12]. The removal of apoptotic germ cells and residual bodies is essential for the completion of spermatogenesis. This task is performed by Sertoli cells; failure can lead to the initiation of autoimmune orchitis [11]. In addition, Sertoli cells also possess inherent immunosuppressive properties due to their ability to secrete indolamin-2,3-dioxygenase (IDO), transforming growth factor beta (TGF-β) and Fas Ligand (FasL), which can even allow the survival of allografts and xenografts [13–15]. Sertoli cells also express inhibitors of complement, apoptosis and granzyme, and can inhibit the proliferation of B and T cells [16, 17]. By expressing FasL, they can induce the death of Fas expressing activated lymphocytes. Additionally, through their secretion of TGF-β and activin A, they can modulate testicular macrophages and T cells to adopt a phenotype that supports immune privilege [17, 18].

An immune privileged environment in the testis is also favored given the high levels of immunoregulatory and immunosuppressive mediators. In this context, TGF-β suppresses immune responses in the testis and has been shown to be secreted by Sertoli and Leydig cells. Sertoli cells produce activin A which can inhibit the production of pro-inflammatory cytokines [17]. Leydig cells contribute to the immune suppressive environment through their secretion of testosterone. Besides regulating spermatogenesis, testosterone differentiates and supports regulatory T cells [19, 20]. Peritubular cells also contribute to this environment by secreting activin A [6, 17].

Importantly, the immune cells present in the testis exhibit immunoregulatory and tolerogenic phenotypes and characteristics [21]. Testicular macrophages, the most abundant testicular cells, are divided into interstitial and peritubular macrophages. Interstitial macrophages, closely associated with Leydig cells, express high levels of immunosuppressive factors such as IL-10 and TGF-β and have a reduced capacity to secrete inflammatory mediators such as TNF, IL-6 and nitric oxide (NO). Peritubular macrophages, located adjacent to seminiferous tubules, express high levels of MHC-II and are thought to induce tolerance via regulatory T cells [21, 22]. Dendritic cells, a minor population in the testis, exhibit immature phenotypes, lack CD80 and CD86 expression, and are incapable of activating lymphocytes under physiological conditions [21, 23]. In addition, the majority of T cells present are regulatory T cells, which are crucial for maintenance of tolerance towards sperm antigens [21].

In summation, several factors and mechanisms come into play to ensure that the germ cells are protected and preserved. Galectins, pleiotropic lectins, are postulated to contribute to this process by their immunoregulatory functions, besides important roles in fertilization.

## Galectins are part of the cellular glycocalyx

A dense, very complex network of sugar moieties connected to proteins and lipids called ‘glycocalyx’ cover every cell in the body. Key components of the glycocalyx are the glycans as the carbohydrates forming sugar conjugates. Despite its critical role in various fundamental cellular processes, its complexity has prevented a deeper understanding of the exact function [24]. Galectins are pivotal in the organization and regulation of the glycocalyx. The creation of galectin-glycan lattices on the cell surface is a coordinated effort involving glycan-modifying enzymes, specifically glycosyltransferases and glycosidases [25, 26].

Galectins belong to an evolutionary highly conserved family of carbohydrate binding lectins that mediate various biological functions and pathologies. Members of the galectin family are pleiotropic molecules involved in physiological cellular processes such as proliferation, differentiation, apoptosis, angiogenesis, wound healing and immune responses. Due to their broad spectrum of cellular distribution and action are they implicated in the development of various diseases including inflammation, infection, autoimmunity, cancer, obesity, cardiovascular diseases or fibrotic remodeling [27–30]. To date, 20 mammalian galectins have been reported, which are divided into three families based on the presence of at least one conserved carbohydrate-recognizing domain (CRD) of about 130 amino acids: (a) *prototypic* containing only one CRD (e.g. galectin-1), (b) *tandem-repeat* with two homologues (but not identical) CRDs (e.g. galectin-9) and (c) *chimera-type* consisting of one CRD connected to an amino-terminal domain (galectin-3) [29]. The CRD recognizes *N-* or *O-*glycans containing N-acetyllactosamine (LacNAc) consisting of galactose (Gal) β1–3/4 linked to N-acetyloglucosamine (GlcNAc) in cell surface glycoproteins and glycolipids or proteoglycans in the extracellular matrix. The binding of galectins to LacNAc is enhanced by increased branching of complex type N-glycans (galectin-1) or repeated LacNAc motifs (galectin-3). In contrast, modifications of the Gal residue e.g. by sialylation (catalyzed by β-galactoside α2,6-sialyltransferase 1 (ST6GAL1) inhibits the binding of galectin-1 (reviewed in [30]).

Although the carbohydrate-dependent binding of spermatozoa to the zona pellucida surrounding the egg highlights a critical role for galectins and glycobiology in mammalian reproduction [31, 32], the functions of galectins in reproductive organs, particularly in males, remain largely unexplored. Among the galectins, galectin-1 and galectin-3 are the best-studied in the testis and epididymis. Therefore, this review aims to summarize the current knowledge on this topic.

## Galectins in testis and epididymis

### Galectin-1

#### Role in homeostasis

The first discovered galectin (galectin-1) represents one of the best-studied and well-characterized galectins in both testis and epididymis. Galectin-1 is involved in a variety of cellular processes, including pathogen recognition, selective induction of Th1 and Th17 apoptosis, inhibition of T cell trafficking, expansion of tolerogenic dendritic and regulatory T cells, maintenance of maternal-fetal tolerance, induction of angiogenesis and suppression of autoimmunity [28, 29, 33–36].

Early reports demonstrated mRNA expression of L14 lectin (identified later as galectin-1) in the adult mouse testis [37]. Subsequent studies analyzing the galectin-1 expression during testis development revealed differential expression patterns in Sertoli cells from embryonic and pre-pubertal testis. During embryonic development before germ cell heterogeneity manifests, a cyclical pattern of galectin-1 expression arises. In the adult testis, Sertoli cells showed a stage-specific cyclical regulation with the highest abundance in spermatogenic stages X-XII and lowest levels at stages VIII-IX [38]. Other studies confirmed the localization in Sertoli cells, but found the protein additionally also in germ and Leydig cells using immunohistochemistry and in testicular extracts by Western blotting [39, 40].

**Fig. 1:**
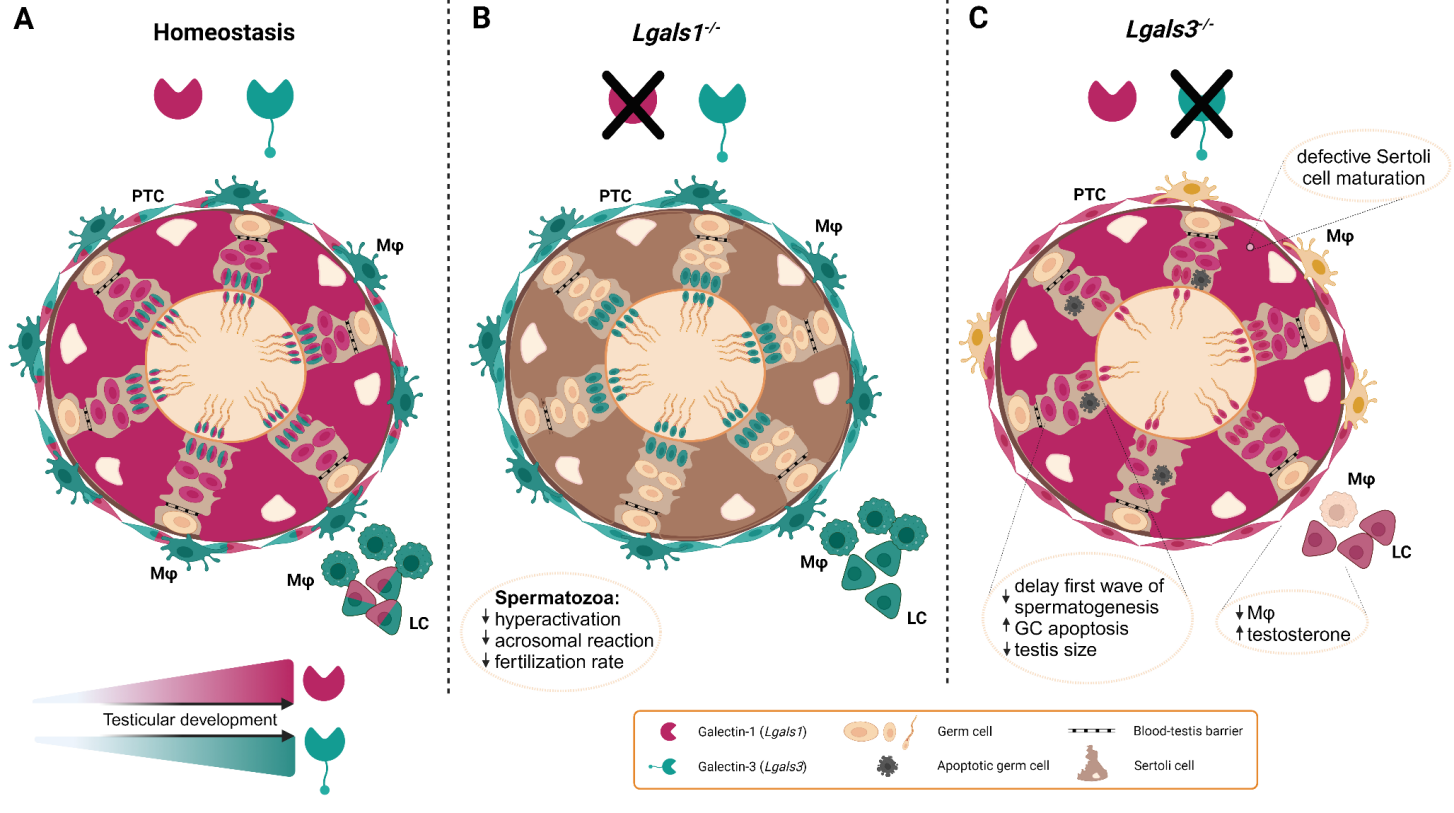
Murine model of testicular galectin-1 and galectin-3 expression patterns and the consequences of *Lgals1* and *Lgals3*depletion on physiological function. **(A)** Color coded cellular expression of galectin-1 (fuchsia color) and galectin-3 (cyan color) in the mouse testis. Galectin-1 and 3 levels increase in an age-dependent manner in the testis and are expressed by peritubular, Leydig and germ cells with galectin-3 only present in later germ cell stages. Sertoli cells express galectin-1, but not galectin-3. In contrast, macrophages show galectin-3, but not galectin-1. **(B)** In *Lgals1*^*-/-*^ mice, sperm functional parameters are affected as indicated. **(C)***Lgals3*^*-/-*^ mice are fertile, however, the testis exhibits impairments as noted in the figure. PTC: peritubular cell, LC: Leydig cell, Mφ: macrophage. Image was created using BioRender

For the rat testis, Dettin et al. confirmed the developmental regulation and stage-specific expression during spermatogenesis for galectin-1 [41]. Here, the testicular concentration of galectin-1 increased in an age-dependent manner in postnatal rats (9–60 days), with similar notions also in porcine and swamp buffalo testis [41–44]. Interestingly, during spermiation (stage VI – VIII) in rat, galectin-1 was localized to the apical part of Sertoli cells as well as on heads of mature spermatids and residual bodies. Following sperm release to the tubular lumen, galectin-1 localization was seen mainly at the basal part of Sertoli cells from where it spread out to the entire cell during progression of germ cell differentiation [41]. A similar pattern of galectin-1 localization was detected in rat Sertoli cells by Özbek et al., however, slight staining differences were observed in the germ cells. Here, additionally positive spermatocytes and round spermatids were recorded [43]. In agreement, our own data demonstrated that in normal rat testis galectin-1 was mainly found in Sertoli and germ cells [45]. The presence of galectin-1 in human Sertoli and germ cells as well as in peritubular cells was reported [46, 47]. Here, a polarized secretion in cultivated Sertoli cells was evident [46] that could occur via extracellular vesicles [48]. In summation (see Table 1 for overview, Fig. 1), reports indicate that the expression of galectin-1 starts in prenatal testis and increases during puberty, when neo-antigen harboring haploid germ cells first appear, a fact that could point to a role in germ cell development, but also in establishing testicular immune privilege.

**Table 1:** The table presents the cellular localization of galectin-1 and galectin-3 in mature testis and epididymis across different species

Galectin type	Organ						Species	Reference
	Testis							
**galectin-1**	GC	SC	PTC	LC	Mφ	Int cells		
	+	+	-				Human	[47]
	+	+	+			+	Human	[46]
	+	+				+	Rat	[41]
	+	+	-	+	-		Rat	[43]
	+	+	-		-		Rat	[45]
		+	+			+	Mouse	[38]
	+	+		+			Mouse	[39]
**galectin-3**	-	-		+			Human	[47]
	-	+					Human	[58]
	-	+		+			Human	[59]
	-	+	+	+	+		Rat	[43]
	+	-	+	+		+	Mouse	[63]
	+	-	+	-	+		Mouse	Own unpublished data
	-	-	+	-	?	+	Boar	[61]
	-	-	+	?	?	+	Bull	[62]
**Galectin**	**Epididymis**						**Species**	**Reference**
	**Initial segment**		**Caput**	**Corpus**		**Cauda**		
**galectin-1**	Muscle cells (ductal wall, vessels)		Muscle cells (ductal wall, vessels)	Muscle cells (ductal wall, vessels)		Muscle cells (ductal wall, vessels)	Rat	[43]
**galectin-3**	Narrow cells, macrophages (interstitial and intraepithelial)		Macrophages (interstitial and intraepithelial)	Principal and basal cells, macrophages (intraepithelial)		Principal and basal cells, macrophages (interstitial)	Rat	[43]
			Connective tissue, Few epithelial cells	Connective tissue, Few epithelial cells		Majority of epithelial cells	Boar	[61]
			Connective tissue, principal and basal cells, sperm	Connective tissue, principal cells		Connective tissue, principal and basal cells	Bull	[62]

Only studies that specify the expression of these galectins at the protein or mRNA level in specific cell types are included. Those that describe expression in the entire organs without distinguishing the positive cell types are excluded. The term ‘interstitial cells’ is used when authors do not specify whether Leydig cells or macrophages are being referred to

The latter is supported by an increasing body of evidence that demonstrates an immunoregulatory function in autoimmune disease models mediated through galectin-1 effects on immune cells [28]. In this context, galectin-1 secreted by murine Sertoli cells co-cultured with dendritic cells has been identified as a factor that induces the differentiation of tolerogenic dendritic cells. This is achieved by decreasing the expression of surface molecules (CD80, CD83, CD86, CCR7, CD11c) crucial for T cell activation and dendritic cell migration to lymph nodes. Furthermore, galectin-1 enhances the secretion of immunoregulatory IL-10 and TGF-β1, while reducing the secretion of pro-inflammatory TNF and IL-12p70 by immature dendritic cells in a dose-dependent manner [49]. By these means, galectin-1 is considered as a crucial mediator of the immunotolerogenic environment created by Sertoli cells in the testis, playing a significant role in maintaining testicular immune privilege. This is well exemplified by the successful application of Sertoli cells in co-transplantation studies (e.g. with pancreatic islets). Additionally, galectin-1 directly inhibits the activation of B and T cells and induces regulatory T cells [18, 50, 51].

In the testicular interstitial space, Leydig cells are both producers and target cells of galectin-1. Stimulation of cultivated Leydig cells from rat with recombinant galectin-1 inhibited testosterone production and caused a reduction in cell viability by eliciting apoptosis [52]. In contrast, the effects of added galectin-1 on growth and cell survival on the mouse MA-10 Leydig cell tumor line were biphasic depending on the concentration. High levels of galectin-1 induced apoptosis by mitochondrial and death receptor pathways, while low concentrations promoted cell proliferation and survival [53].

In relation to the presence and function of galectin-1 in the epididymis, the available data is very scarce. In rat epididymis, the presence of galectin-1 was demonstrated by immunohistological staining in the smooth muscle layers surrounding the epididymal ducts and in the blood vessels, while the epithelial cells were negative [43]. In murine epididymis, the expression of galectin-1 was investigated in protein extracts from all regions revealing a gradual increase from caput to distal parts (cauda). Moreover, galectin-1 was detected in fresh epididymal spermatozoa from cauda, but not from caput suggesting transfer from epithelial secretions and a role in sperm maturation This is supported by the presence of soluble galectin-1 in the epididymal fluid from cauda [40]. Galectin-1 was also found in human seminal plasma [54, 55].

Functional investigations on the role of galectin-1 in testis and epididymis were mainly derived from mice deficient in *Lgals1* (Fig. 1). Caudal spermatozoa from *Lgals1*^*−/−*^ mice exhibited defects in acquiring hyperactivation, an important step required for fertilization, i.e. the penetration of the egg coat. Moreover, *Lgals1*^*−/−*^ sperm revealed a decreased ability for a progesterone-induced acrosome reaction, another process necessary to enter the egg. Mechanistically, this was related to a deficiency in sperm membrane potential hyperpolarization. Named effects were rescued by addition of exogenous recombinant galectin-1. These in vitro data are supported by lower fertilization rates in *Lgals1*^*−/−*^ mice compared to wild type counterparts [40], emphasizing the relevance for galectin-1 in sperm function and fertilization. Of note, in contrast to the mouse, spermatozoa from rat cauda epididymidis showed very strong galectin-1 immunostaining around the plasma membrane of the head indicating species-specific differences in the presence of this lectin in spermatozoa from different regions of the epididymis [41].

#### Role in pathological conditions

Since galectin-1 is involved in the modulation of immune responses and shows anti-inflammatory properties in autoimmune diseases, investigations on the role of galectin-1 were prompted in a mouse model of experimental autoimmune epididymo-orchitis (EAEO). EAEO is a well-established rodent model of autoimmune-based inflammation mimicking pathology observed in the human testis and epididymis that ultimately can lead to infertility [51]. The pathology in the testis is characterized by production of pro-inflammatory and pro-fibrotic mediators (e.g. TNF, CCL2, activin A), immune cell infiltration, disruption of spermatogenesis, thickening of the lamina propria likely contributing to loss of the adluminal compartment and subsequent aspermatogenesis. Later stages of the disease show disruption of the BTB, extensive necrosis in seminiferous tubules and fibrotic remodeling [51, 56]. Our own data demonstrated that in the rat EAEO testis total expression of galectin-1 was downregulated, an observation likely based on the concomitant loss of galectin-1 expressing germ cells. Primary rat Sertoli cells demonstrated increased production of galectin-1 after challenge with TNF, a cytokine elevated in EAEO testis. Moreover, galectin-1 enhanced TNF-stimulated production of *Il1a*, *Il6* and *Ccl2* through MAP kinase activation in Sertoli cells [45]. In contrast, in a murine model of EAEO, the protein expression of this lectin was unchanged [39].

Induction of EAEO in *Lgals1*^*−/−*^ mice led to a significant reduction in the incidence and severity of the disease as compared to wild type, showing only focal tubular damage and lower number of apoptotic germ cells. Surprisingly, these effects are opposite to established inhibitory function of endogenous galectin-1 in other immune privileged site such brain as documented by high susceptibility of *Lgals1*^*−/−*^ mice to development of experimental autoimmune encephalomyelitis [35]. However, the role of galectin-1 in this model seems to be context- and cell-dependent as intraperitoneal administration of exogenous recombinant galectin-1 during the course of EAEO significantly reduced the severity of the disease. Therefore, a dual role of endogenous and exogenously administered galection-1 is evident in the control of autoimmune-based testicular inflammation [39].

Interestingly, under normal conditions testicular macrophages do not express galectin-1, while in EAEO testis, CD68^+^ macrophages located near granulomas show galectin-1 expression. It is plausible that galectin-1 secreted by newly arriving macrophages is implicated in enhanced TNF induced inflammatory effects in Sertoli cells as mentioned above [45].

Galectin-1 binds to LacNAc on branches of *N-* and *O*-glycans, which are synthesized by the synchronized activity of glycosyltransferases. There are three important post-translational mechanisms to form galectin-1 binding sites including: (a) activity of core 2 glucosaminyl (N-acetyl) transferase 1 (Gcnt1) for synthesis of core 2 *O*-glycans, which are the backbone of galectin-1 ligands, (b) suppression of ST6 beta-galactoside α-2-6-sialyltransferase 1 (St6gal1) activity, that abrogates galectin-1 binding to some terminal *N*-acetylglucosamines by adding α-2-6-sialic acid and (c) branching of *N*-glycans by mannosyl (α-1,3-)-glycoprotein ß-1,2-N-acetylglucosaminyltransferases (Mgat) like Mgat5 [57]. The experiments demonstrated that transcripts of *St6gal1* were upregulated with a concomitant downregulation of *Mgat5* in rat EAEO testis. In line with these findings, the binding of Sambucus nigra agglutinin, recognizing terminal α-2-6 sialic acid residues, was increased in EAEO testis. From a mechanistic perspective, the in vitro binding of galectin-1 to Sertoli and peritubular cells following an inflammatory challenge with TNF was reduced due to changes in the glycan composition. These findings indicate that α-2-6-sialylation of *O*- and *N*-glycans increases in inflamed testis and galectin-1 modulates inflammatory responses in Sertoli cells by enhancing the pro-inflammatory activity of TNF [45].

In seminal plasma, the levels of galectin-1 were significantly elevated in azoospermic patients [54, 55].

### Galectin-3

#### Role in homeostasis

Galectin-3 is the only member of the chimera type of galectins, which consists of one CRD connected to the amino-terminal domain that mediates oligomerization into pentamers [29]. Intracellularly, galectin-3 is localized in the nucleus and cytoplasm, but is also found outside of the cell. Galectin-3 influences different inflammatory processes through its interaction with certain immune cell populations such as neutrophils, monocytes or macrophages, thereby facilitating their recruitment and migration to the inflammatory site. It also interacts with a variety of cell types involved in wound healing including monocytes, macrophages, neutrophils, keratinocytes, and fibroblasts (reviewed in [28]).

However, the testicular or epididymal function of galectin-3 is largely unknown. Galectin-3 expression at the protein and gene levels were found in the testes of human, boar, bull, rat and mouse [58–62].

In the healthy testis, Sertoli cells, germ cells, peritubular cells and macrophages mainly express galectin-3. Similar to galectin-1, the expression levels of galectin-3 increases during testicular development [43, 58, 59]. In rat, at postnatal day (PND) 5 before puberty, only macrophages were positive for galectin-3, with peritubular cells following from PND 20 after commencement of spermatogenesis. Sertoli and Leydig cells were seen positive both at PND 50 and 70 [43].

Here, it needs to be mentioned that some species-specific differences in galectin-3 expression were reported (Table 1). In relation to cellular localization of galectin-3 in the testis, species specific differences were noted for mouse, bull and boar as compared to rat and human testis (summarized in Table 1) ([43, 47, 58, 59, 61–64], own unpublished data). In contrast to rat and human, Sertoli cells from mouse, bull and boar do not express galectin-3 and in these species the presence of this lectin is limited to peritubular cells, macrophages and/ or late stages of germ cells (Table 1). In cultivated porcine Sertoli cells galectin-3 expression was stimulated in a dose-dependent manner by FSH, epidermal growth factor and TNF [58], while in Leydig cells galectin-3 is involved in the regulation of steroidogenesis and secretion of testosterone. Depletion of galectin-3 in murine Leydig cells resulted in increased expression of enzymes crucial for testosterone biosynthesis, such as *Cyp11a1*, *Hsd3b1*, and *Cyp17a1*. This upregulation was associated with elevated secretion of testosterone and androstandiol [65].

Analysis of the physiological function of galectin-3 built largely on constitutive knock-out mice for galectin-3 (*Lgals3*^*−/−*^). In this context, *Lgals3*^*−/−*^ mice showed (i) a delay in the first wave of spermatogenesis, (ii) decreased number of germ cells at PND 5 and 15 as well as (iii) an impaired maturation of Sertoli cells. Although adult animals are fertile, the testicular weight was significantly reduced compared to age-matched wild type controls. To determine the causes of testicular atrophy, testicular apoptosis was monitored and demonstrated an elevated rate of germ cell apoptosis due to a perturbed Bcl-2, Bax and Bak expression ratio, molecules responsible for live/death balance in cells. Interestingly, the expression of enzymes involved in steroid biosynthesis as well as levels of serum testosterone were increased in *Lgals3*^*−/−*^ mice. Moreover, galectin-3 seems to play a role in the control of the testicular macrophage population since the number of F4/80^+^CD11b^+^ macrophages within the population of CD45^+^ immune cells was reduced in *Lgals3*^*−/−*^ testis [65]. An apoptosis-related function of galectin-3 in the testis was highlighted in studies showing upregulated expression of galectin-3 in the testes of Nucling-deficient mice. Nucling interacts with galectin-3 and mediates apoptosis by inhibiting its expression via NF-κB signaling [66]. Altogether, the studies point to an important role of galectin-3 in the regulation of spermatogenesis, steroidogenesis and maintenance of the testicular macrophage population.

In the epididymis and ejaculate, galectin-3 is present in epididymal cells (narrow, basal, principal cells), ejaculated spermatozoa and extracellular vesicles in human seminal plasma [60–62]. Expression levels of galectin-3 were seen lower in caput than in the distal parts (corpus, cauda) of the epididymis in human, bull and rat [43, 61, 62]. In development, at PND 5 in all parts of the epididymis (initial segment, caput, corpus and cauda) only interstitial macrophages in the connective tissue between the epididymal duct were positive for galectin-3, while at PND 20, intraepithelial macrophages additionally acquired expression. During adulthood (PND 50 and 70), in addition to macrophages, narrow cells in the initial segment, as well as principal and basal cells in the corpus and cauda epididymidis, began to express galectin-3 [43]. A similar expression pattern was observed in the bull epididymis. Interestingly, bull spermatozoa, which were negative for testicular galectin-3, acquired its expression in the caput epididymis and subsequently lost it during their passage through the corpus and cauda [62]. In combination, these data point to a role of galectin-3 in epididymal sperm maturation, but it needs to be noted that firm evidence is lacking. Variations in the galectin-3 presence in the immature and mature testis and epididymis may be related to the hormonal status, progression of spermatogenesis, expression of neo-antigens on the surface of spermatozoa and development of immune tolerance to haploid germ cells.

#### Role in pathological conditions

Galectin-3 has been discussed as a marker of aggressiveness in testicular tumors, with *LGALS3* transcripts down-regulated in malignant Sertoli cell tumors, but seen increased in non-seminomatous testicular germ cell tumors [59]. In human biopsies obtained from infertile men with different diagnosis of testicular impairment (Sertoli cell only syndrome (SCOS), arrest of spermatogenesis at the meiotic or spermatid stage) an increased expression of galectin-3 was accompanied by transfer of the protein in Sertoli cells from the cytoplasm to the nucleus [58]. In line with these data, nuclear staining of Sertoli cells in SCOS was also reported in earlier studies by Wollina et al. [47]. In the serum and seminal plasma from azoospermic patients the levels of galectin-3 were significantly upregulated as compared to fertile men [55].

### Other galectins

In a recently published study, the expression of galectin-9 was for the first time reported on the surface of testicular immune cells (CD3^+^, CD4^+^, CD8^+^ T cells, NK cells) from mice using flow cytometry measurements [67]. Galectin-9 is a ligand for the T-cell immunoglobulin and mucin-domain containing-3 (TIM-3) receptor. Both, in mouse and human, binding of galectin-9 to TIM-3 leads to apoptosis of Th1 and Th17 cells and induces immune tolerance [30]. Furthermore, galectin-7 was postulated as a marker of immature Sertoli cells [68].

## Conclusions and future perspectives

The expression patterns, functions and mechanisms of galectins in the male reproductive tract are not yet fully discovered. However, accumulating evidence suggest that galectin-1 acts as a pluripotent regulator of the immune system and is highly expressed in immune privileged organs such as the placenta, ovary and testis. Given that its levels increase at puberty in the testis, it can be postulated that galectin-1 could function in regulating immune privilege, but also sperm functions. The role of galectin-1 in inflammatory and pathological conditions is highly context dependent. A major influence of its action is derived from modifications of the pattern of *N-* and *O*-glycans that permits or inhibits binding of galectin-1 to target cells. Detailed mechanistic studies focusing on the intracellular signaling pathways through which galectin-1 transmits its functions in regulating spermatogenesis and testicular immunity are far from being understood. In comparison to galectin-1, the role of galectin-3 in the testis and epididymis is much less well explored. Initial studies point to a function in regulating spermatogenesis and steroidogenesis, a role that may – at least partly - be mediated by the observed targeting of Sertoli cells and testicular macrophages. Exploring how galectin-3 modulates interaction between immune cells and testicular cells in normal and pathological condition, specifically autoimmune settings is urgently needed. In general, galectins remain to be investigated in detail in the epididymis. Last but not least, by identifying the possible interaction of galectins with other molecules such as cytokines, chemokines and extracellular mediators in the testicular and epididymal microenvironment under physiological and pathological conditions can help in delineating their exact functions. This would include elucidating whether galectins play a role in the establishing the niches that have recently been uncovered for macrophages in the testis and epididymis.

## Data Availability

Data sharing not applicable to this article as no datasets were generated during the current study.
